# Safranal Alleviates Dextran Sulfate Sodium-Induced Colitis and Suppresses Macrophage-Mediated Inflammation

**DOI:** 10.3389/fphar.2019.01281

**Published:** 2019-11-01

**Authors:** Peeraphong Lertnimitphun, Yiwen Jiang, Nami Kim, Wenwei Fu, Changwu Zheng, Hongsheng Tan, Hua Zhou, Xue Zhang, Weizhong Pei, Yue Lu, Hongxi Xu

**Affiliations:** ^1^School of Pharmacy, Shanghai University of Traditional Chinese Medicine, Shanghai, China; ^2^Institute of Cardiovascular Disease of Integrated Traditional Chinese and Western Medicine, Shuguang Hospital, Shanghai University of Traditional Chinese Medicine, Shanghai, China; ^3^Shanghai Traditional Chinese medicine Co., Ltd., Shanghai, China

**Keywords:** *Crocus sativus*, saffron, safranal, macrophages, MAPKs, NF-κB, traditional Chinese medicine, colitis

## Abstract

**Introduction:** *Crocus sativus* (saffron) is widely used in China, Iran, and India for dyeing and as a food additive and medicinal plant. Safranal, as one of the main constituents of saffron, is responsible for its aroma and has been reported to have anticancer, antioxidant, and anti-inflammation properties.

**Objective:** In this study, we investigated the anti-inflammatory effects of Safranal in RAW264.7 cells, bone marrow-derived macrophages (BMDMs), and dextran sulfate sodium (DSS)-induced colitis mice.

**Methods:** Safranal toxicity was determined using an MTT assay. We evaluated the inhibitory effect of nitric oxide (NO) and levels of inducible nitric oxide synthase (iNOS) and cyclooxygenase-2 (COX-2) in RAW264.7 cells and BMDMs. We assessed the inhibitory effect of pro-inflammatory cytokines, and the mRNA expressions of interleukin-6 (IL-6), tumor necrosis factor alpha (TNF-α), classical inflammatory pathways (MAPK and NF-κB), and the nuclear translocation factors AP-1 and NF-κB p65 were investigated. The *in vivo* anti-inflammatory effects of Safranal were assessed in a DSS-induced colitis model. DSS3.5% was used to induce colitis in mice with or without Safranal for 7 days; weight and disease activity index (DAI) were recorded daily. At the end of the experiment, the colon, mesenteric lymph nodes (MLNs), and spleen were collected for flow cytometry, ELISA, and Western blot analysis.

**Results:** Safranal suppressed NO production, iNOS, and COX-2 in lipopolysaccharide (LPS)-stimulated RAW264.7 cells and BMDMs. Safranal decreased the production and mRNA expression of IL-6 and TNF-α in the RAW264.7 cell line and inhibited the phosphorylation and nuclear translocation of components of the MAPK and NF-κB pathways. Safranal alleviated clinical symptoms in the DSS-induced colitis model, and colon histology showed decreased severity of inflammation, depth of inflammatory involvement, and crypt damage. Immunohistochemical staining and flow cytometry showed reduced macrophage infiltration in colonic tissues and macrophage numbers in MLNs and the spleen. The levels of colonic IL-6 and TNF-α also decreased in Safranal-treated colitis mice. This study elucidates the anti-inflammation activity of Safranal, which may be a candidate for inflammatory bowel syndrome (IBD) therapy.

## Introduction

Saffron (*Crocus sativus*) is a well-known spice that is widely used throughout the Middle East and Asia as a food additive, drinks, dye, and medicinal plant in China. Due to its rarity, labor-intensive production, and growing demand, the price of saffron has increased in the past few years. Saffron is used in traditional Chinese medicine for anxiety and cardiovascular diseases in China. Compounds derived from saffron, such as Safranal, Crocin, Crocetin, and Picrococin are responsible for the color, aroma, and flavor of saffron. Safranal is responsible for the unique aroma of this spice, and it may be synthesized for batch production. Safranal alleviates myocardial ischemia–reperfusion (IR) injury (Bharti et al., 2012) and protects against rotenone-induced neurotoxicity associated with the Nrf2 signaling pathway, which supports use of Safranal as a therapeutic drug for the treatment of Parkinson’s disease (Pan et al., 2016). Safranal has shown anticancer activity *via* inducing cell death in HeLa and MCF7 cancer cell lines (Malaekeh-Nikouei et al., 2013). However, its mechanisms and use are unclear and must be further investigated.

Macrophage functions include pro-inflammatory mediators production and increasing inflammatory response, leading to many inflammatory diseases, such as inflammatory bowel disease (IBD) (Eissa et al., 2018). Ulcerative colitis (UC) is an IBD that relapses. UC is characterized by weight loss, diarrhea, abdominal pain, and rectal bleeding (Petryszyn and Paradowski, 2018). UC affects patients’ quality of life, and UC may lead to colonic cancer if left untreated (Neurath, 2019). Though first-line drugs, such as immunotherapies and steroids, are effective, but the side effects and relapse rate of UC patients are high (Lucidarme et al., 2019). Some patients do not respond to first-line drugs, such as TNF-α inhibitors (Weisshof et al., 2019). Thus, alternative drugs are needed to be investigated. The pathological characteristics of UC include depletion of the epithelial barrier, which allows colonic immune cells to interact with colonic bacteria and induce inflammatory responses (Du et al., 2015). Recent studies demonstrate that innate immune cells, such as macrophage infiltration and activation, increase the severity of colitis (Yan et al., 2018). Of the active compounds from saffron, Crocin has showed promising effect in the treatment of colitis (Rezaei et al., 2019), but the effect of Safranal on colitis has not been investigated. The present study investigated the anti-inflammatory effects of Safranal in RAW264.7 cells, bone marrow-derived macrophages (BMDMs), and dextran sulfate sodium (DSS)-induced colitis.

## Materials and Methods

### Animals and the Induction of Experimental Colitis

Female BALB/c mice (18–20 g) were purchased from the Shanghai SLAC Laboratory (Shanghai, China) and housed in an SPF (specific pathogen-free) and temperature-controlled (25 ± 2°C) environment with a 12-h light/dark cycle in the Shanghai University of Traditional Chinese Medicine. Mice were provided with normal diet and drinking water. Experiment began after mice adapted to the new environment for at least 1 week before the beginning of the experiment. To induce colitis, mice were given DSS (MW 36000-50000, MP Biomedical, CA, USA) in drinking water (3.5%, *v*/*w*) *ad libitum* for 7 days. Mice were randomly divided equally (*n* = 10) into four groups: normal control group (given only food and water without DSS), DSS model group (administered DSS in drinking water), low-concentration Safranal group (administered DSS in drinking water and 200 mg/kg, p.o.), and high-concentration Safranal group (administered DSS in drinking water and 500 mg/kg, p.o). Weight and disease activity index (DAI) were recorded daily. Mice were euthanized after 7 days, and the colon, mesenteric lymph nodes (MLNs), and spleen were harvested for further analyses. The DAI includes weight loss (0, none; 1, 0–5%; 2, 5–10%; 3, 10–20%; 4, > 20%), stool consistency change (0, none; 1 and 2, loose stool; 3 and 4, diarrhea), and bleeding (0, none; 1, trace of fecal occult blood; 2, mild occult blood; 3, obvious occult blood; 4, gross bleeding) (Zhang et al., 2019).

### Cell Culture and Activation of Macrophages

The RAW264.7 cell line was purchased from the cell bank of Shanghai Institute of Cell Biology and Biochemistry, Chinese Academy of Science (Shanghai, China). Cells were cultured in Dulbecco’s modified Eagle’s medium (DMEM) containing 10% fetal bovine serum, penicillin, and streptomycin (100 U/ml). Cells were incubated in 37°C and 5% CO_2_ overnight before pretreatment with various concentrations of Safranal for 1 h prior to lipopolysaccharide (LPS) stimulation (1 µg/ml).

BMDMs were isolated from 6- to 8-week-old BALB/c mice as previously described (Zhao et al., 2019). In brief, monocytes from bone marrow were harvested from the hind legs via flushing with DMEM, and cells were supplied with 70% L929-conditioned DMEM containing 10% fetal bovine serum (FBS) penicillin and streptomycin (100 U/ml) at a density of 5 × 10^5^ cells/ml. Cell culture media were changed every 2 days for 1 week before experiments. To stimulate BMDMs, Safranal was added 1 h prior to LPS stimulation (10 µg/ml) and IFN-γ (20 ng/ml). The L929 cell line was purchased from the cell bank of Shanghai Institute of Cell Biology and Biochemistry, Chinese Academy of Science (Shanghai, China). The L929 cell line was cultured at a density of 5 × 10^5^ cells/ml in DMEM containing 10% fetal bovine serum, penicillin, and streptomycin (100 U/ml) for 5 days before collecting the supernatants for L929-conditioned DMEM.

### Cell Viability Assay

Cell viability was determined using the MTT (3-(4,5-dimethylthiazol-2-yl)-2,5-diphenyltetrazolium bromide) assay. RAW264.7 cells were seeded in 96-well plates at a concentration of 1 × 10^5^ cells/ml overnight. Cells were treated with various concentrations of Safranal for 24 h before the addition of MTT (0.5 mg/ml) and incubated for 4 h. The supernatant was removed and hydrochloride–isopropanol was added to the precipitate and mixed until fully dissolved. The optical density (OD) was measured at 570 nm.

### Measurement of NO Production

Cells were seeded at 5 × 10^5^ cells/ml overnight and incubated with or without Safranal (10 or 50 µM) for 1 h. Cells were stimulated with LPS for 24 h at 37°C and 5% CO_2_ in an incubator. Supernatants were collected and analyzed in a Griess Reagent System, as described in the manufacturer’s instructions (Promega, WI, USA).

### Measurement of IL-6 and TNF-α

RAW264.7 cells were seeded at 5 × 10^5^ cells/ml overnight. Safranal (10 or 50 µM) was added for 1 h, and cells were stimulated with LPS for 24 h at 37°C and 5% CO_2_ in an incubator. Supernatants were collected and centrifuged at 3,000 rpm for 5 min at 4°C for analyses of IL-6 and TNF-α concentrations using ELISA (R&D systems, Minneapolis, MN, USA), according to the manufacturer’s instructions.

### Quantitative Real-Time PCR

RAW264.7 cells were seeded at 5 × 10^5^ cells/ml and grown overnight. Safranal (10 or 50 µM) was added for 1 h, and cells were stimulated with LPS for 8 h at 37°C and 5% CO_2_ in an incubator. RNA was extracted using TRIzol (TaKaRa, Kusatsu, Shiga, Japan) followed by synthesis of cDNA *via* a reverse transcriptase (TaKaRa, Kusatsu, Shiga, Japan). Quantitative RT-PCR analyses were performed using SYBR Green (TOYOBO, Osaka, JAPAN), and the results were analyzed using the 2^−ΔΔCT^ method and normalized to β-actin expression. The following primers were used: mouse IL-6 forward, 5′-CTGCAAGAGACTTCCATCCAGTT-3′, IL-6 reverse, 5′-GAAGTAGGGAAGGCCGTGG-3′; mouse TNF-α forward, 5′-CGAGTG ACAAGCCTGTAGC-3′, TNF-α reverse, 5′-GGTGTGGGTGAGGAGCACAT-3′; and mouse β-actin forward, 5′-TCAGCAATGCCTGGGTACAT-3′, mouse β-actin reverse, 5′-ATCACTATTGGCAACGAGCG-3′.

### Western Blot Analysis

RAW264.7 cells and colon tissues were homogenized, and protein levels were quantified using BCA reagent (Beyotime, Shanghai, China). Nuclear and cytoplasmic extractions were performed as instructed by the manufacturer (Beyotime, Shanghai, China). Samples were loaded and electrophoresed in 10–12% SDS-PAGE and then transferred to nitrocellulose membranes. Membranes were blocked in 5% nonfat milk diluted in TBS-T for 2 h followed by incubation with primary antibodies. The following primary antibodies were used in this experiment: iNOS, COX-2, phospho-IKKα/β, IKKα/β, phospho-IκBα, IκBα, phospho-ERK ½, ERK ½, phospho-p38, p38, p65, phospho-c-Jun, c-Jun, c-Fos, β-tubulin, Lamin A/C, phospho-JNK, JNK, IRAK4, and β-actin at 1:1,000 dilution (Cell Signaling Technology, Danvers, MA, USA). Secondary antibodies included horseradish peroxidase (HRP)-conjugated goat anti-rabbit and anti-mouse IgG at 1:2,500 dilution.

### Immunofluorescence

RAW264.7 cells were seeded on slide cover glass at 5 × 10^5^ cells/ml, fixed in 4% paraformaldehyde for 15 min, and washed with phosphate-buffered saline (PBS). Triton-X-100 (0.1%) was used to increase cell permeability. Cells were washed with PBS before blocking with 3% BSA. Cells were incubated overnight with primary antibodies p65, phospho-c-Jun, and c-Jun at 1:400, 1:100, and 1:6,400 dilution, respectively (Cell Signaling Technology, Danvers, MA, USA) and incubated with a fluorescein isothiocyanate (FITC)-conjugated anti-rabbit IgG antibody for 1 h. DAPI (SouthernBiotech, AL, USA) was used to stain nuclei, and fluorescence microscopy (Olympus, Tokyo, Japan) was used to capture images.

### Flow Cytometry

Flow cytometry was performed as previously described (Simovic Markovic et al., 2016). In brief, spleen and MLNs from DSS-induced colitis mice were homogenized. Spleen cells were incubated with Red Blood Cell Lysis Buffer (Beyotime, China), and the reaction was stopped with the addition of PBS. MLNs and spleen cells were incubated with anti-mouse CD11b (eBioscience, CA, USA) and anti-mouse F4/80 (eBioscience, CA, USA) antibodies for 30 min and washed with PBS twice before analysis in a CytoFlex FCM (Beckman Coulter, USA). Data were analyzed using CytExpert software.

### H&E Staining and Histological Scoring

The colon was fixed in 4% phosphate-buffered paraformaldehyde, embedded in paraffin, cut into sections, and placed on microscope slides. Slides were stained with hematoxylin and eosin (H&E) and observed using a DP-72 microscope (Olympus, Tokyo, Japan) to evaluate histological damage. Histological scoring was based on three parameters as described below (Lee et al., 2015): a) severity of inflammation: 0 = no inflammation; 1 = mild; 2 = moderate; 3 = severe; b) depth of inflammatory involvement: 0 = no inflammation; 1 = mucosa; 2 = mucosa and submucosa; 3 = transmural; and c) crypt damage: 0 = intact crypts; 1 = loss of the basal one-third; 2 = loss of the basal two-thirds; 3 = entire crypt loss and changes in the epithelial surface with erosion.

### Immunohistochemistry

For immunohistochemical (IHC) staining, mouse anti-rabbit F4/80 (GB11027, Servicebio, Hubei, China, 1:300 dilution) was used as the primary antibody, and goat anti-rabbit IgG was the secondary antibody (GB23303, Servicebio, Hubei, China, 1:200 dilution). IHC was performed using previously described protocols (Dong et al., 2019). Negative control sections were treated in the same manner, with omission of the primary antibody. Images were captured using a DP-72 microscope (Olympus, Tokyo, Japan). The number of macrophages present in colonic tissues were quantified using Image Pro-Plus v.6.0 software *via* analyses of the mean value of integral optical density (IOD).

### Drug and Reagents

Safranal was purchased from Sigma Aldrich (MO, USA), with a purity of ≥90.0%. DMEM, FBS, penicillin, and streptomycin were purchased from Gibco (Grand Island, NY/Carlsbad, CA, USA). *Escherichia coli* LPS was obtained from Sigma Chemical Co. (MO, USA). Milli-Q water was supplied from a water purification system (Millipore, MA, USA). The HRP-conjugated goat anti-rabbit IgG was purchased from Invitrogen (Carlsbad, CA, USA). DSS (molecular weight of 36,000–50,000 Da) was obtained from MP Biomedicals (Santa Ana, CA, USA).

### Statistical Analysis

Data were statistically analyzed and the results are presented as the mean ± SEM deriving from at least three independent experiments. Statistical analyses were performed using GraphPad Prism Software 5.0 (San Diego, CA, USA). Differences between two groups were analyzed using an unpaired Student’s *t* test, whereas multiple comparisons were assessed using one-way analysis of variance (ANOVA) with Tukey’s multiple comparison test. *P* < 0.05 was considered statistically significant.

## Results

### Safranal Inhibits NO Production and iNOS and COX-2 Expression in RAW264.7 Cells and BMDMs

**Figure 1:**
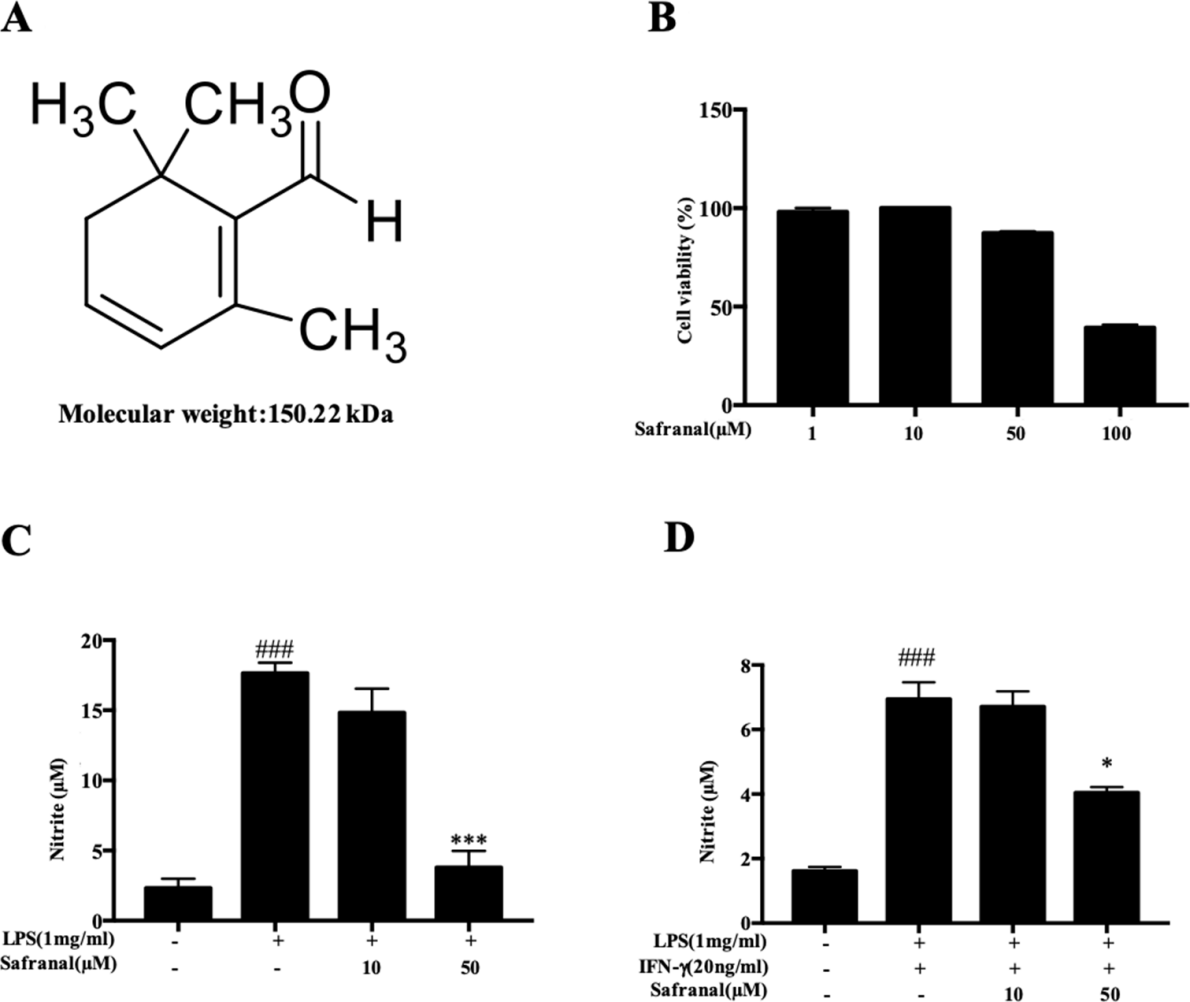
Safranal inhibits NO production in RAW264.7 cells. **(A)** Chemical structure of Safranal. **(B)** Effect of Safranal on cytotoxicity in RAW264.7 cells. Cells were treated with various concentrations of Safranal for 24 h, and cell viability was measured using the MTT assay. **(C)** Effect of Safranal on NO production. RAW264.7 cells were incubated with lipopolysaccharide (LPS, 1 μg/ml) alone or together with various concentrations of Safranal. After 24 h, the culture medium was collected for measurement of NO production using Griess reagent. **(D)** The effect of Safranal on NO production. Bone marrow-derived macrophages were incubated with LPS (1 μg/ml) and IFN-γ (20 ng/ml) or various concentrations of Safranal. After 24 h, the culture medium was collected for measurement of NO production. The data shown are representative of three independent experiments. Data are the means ± SEM of three independent experiments. ^###^*p* < 0.001 compared to non-treated group, *p < 0.05 compared to LPS, ***p < 0.001 compared to LPS.

**Figure 2:**
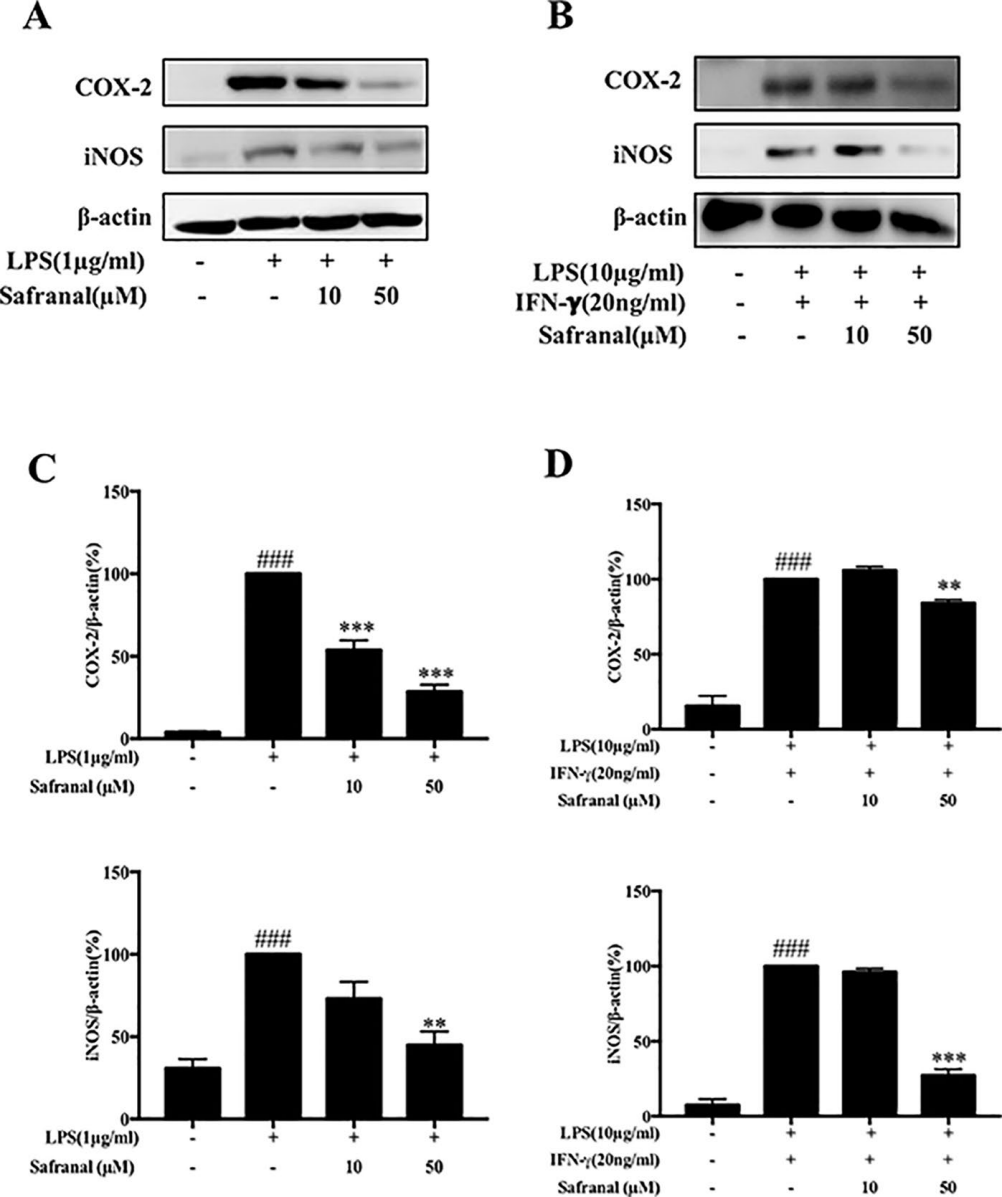
Safranal inhibits iNOS and COX-2 expression in RAW264.7 cells and bone marrow-derived macrophages (BMDM). Macrophages were incubated at 5 × 10^5^ cells/ml in 12-well plates and incubated with Safranal 1 h prior to stimulation. After 24 h, cells were lysed, and total protein lysates were collected for Western blot analyses. **(A)** RAW264.7 cells and **(B)** BMDMs. Relative density of proteins was normalized to β-actin **(C**, **D)**. The data shown are representative of three independent experiments. Data are expressed as the means ± SEM. ^###^*p* < 0.001 compared to non-treated group, **p < 0.01 compared to LPS, ***p < 0.001 compared to LPS.

To evaluate the cytotoxicity of Safranal in RAW264.7 cells, the MTT assay was performed. After treatment with various concentrations of Safranal (**Figure 1A**) for 24h, the highest suitable concentration of Safranal for *in vivo* study was 50 µM (**Figure 1B**). Therefore, the following experiments were performed using 50 µM Safranal. Nitric oxide is the product of LPS-stimulated macrophages, which is crucial in the inflammatory response (Takahashi et al., 2019). Hence, we investigated the ability of Safranal to inhibit NO production and found that Safranal inhibited NO production of RAW264.7 cells and BMDMs after 24 h stimulation (**Figures 1C, D**). As the production of NO is related to iNOS and COX-2 enzymes, we applied Western blot analyses to detect the levels of iNOS and COX-2 proteins. The results showed that Safranal dose-dependently decreased iNOS and COX-2 levels in both RAW264.7 cells and BMDMs (**Figures 2A–D**). Taken together, these results indicate that Safranal exhibits potential anti-inflammatory properties in a dose-dependent manner.

### Safranal Inhibits the Production and mRNA Expression of IL-6 and TNF-α In RAW264.7 Cells

**Figure 3:**
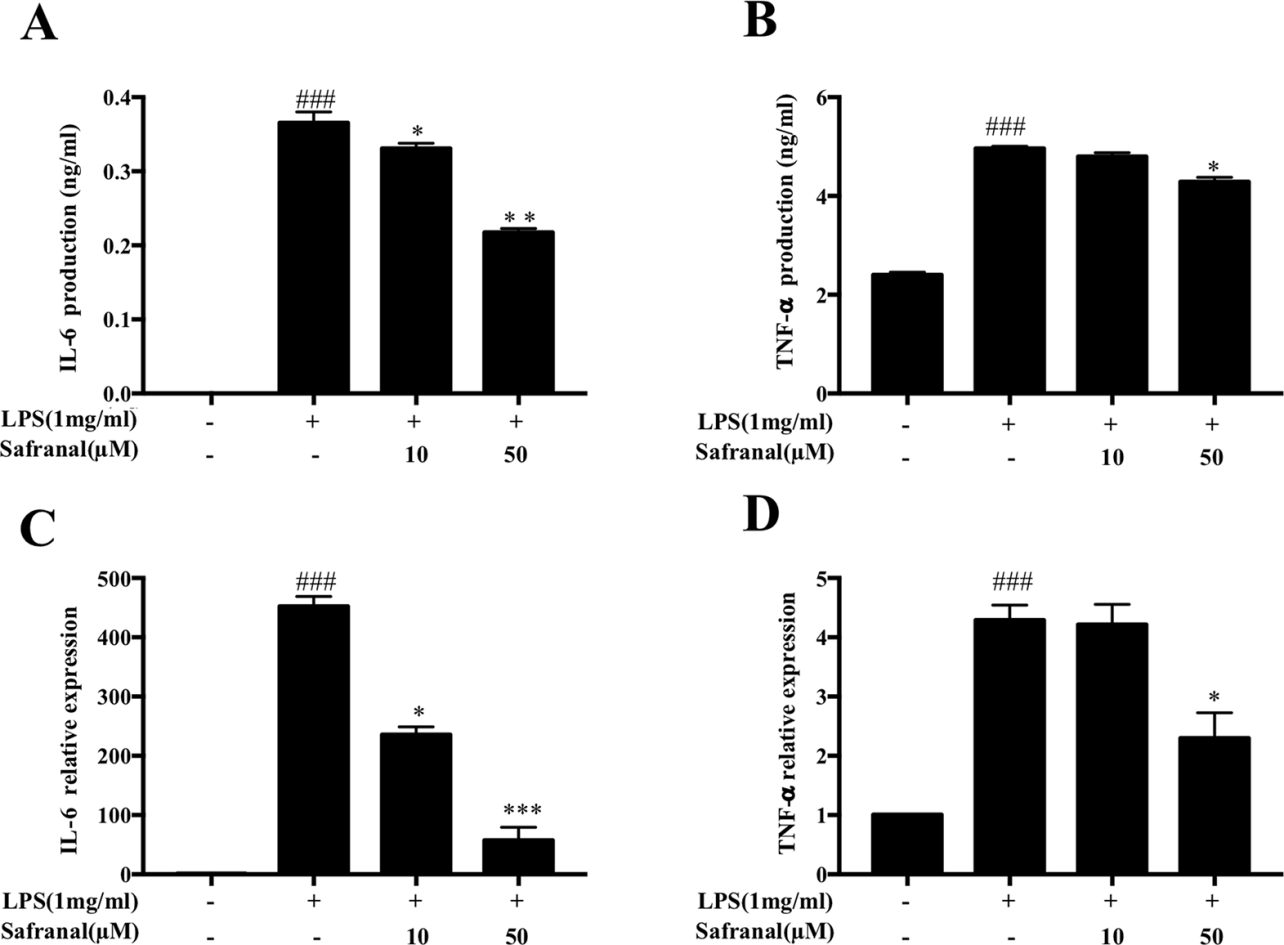
Safranal inhibits cytokine IL-6 and TNF-α production and mRNA expression in lipopolysaccharide (LPS)-stimulated RAW 264.7 cells. Cells were cultured at 5 × 10^5^ cells/ml and incubated with Safranal for 1 h followed by stimulation with LPS (1 μg/ml) for 24 h. **(A)** IL-6 and **(B)** TNF-α in cell supernatants were assayed using ELISA kits. mRNA expression of **(C)** IL-6 and **(D)** TNF-α was analyzed using RT-PCR. The data shown are representative of three independent experiments and indicate the means ± SEM. ^###^*p* < 0.001 compared to non-treated group, *p < 0.05 compared to LPS, **p < 0.01 compared to LPS, ***p < 0.001 compared to LPS.

When activated by Gram-negative bacteria LPS, macrophages secrete pro-inflammatory cytokines, such as IL-6 and TNF-α (Ran et al., 2018), to enhance the inflammatory response. We investigated the effects of Safranal on cytokine production of stimulated RAW264.7 cells. Cells were pretreated with Safranal for 1 h and stimulated with LPS. Supernatants were collected for ELISA, whereas the cells were collected for reverse transcriptase PCR (RT-PCR). The results showed that Safranal significantly reduced the production (**Figures 3A, B**) and mRNA expression (**Figures 3C, D**) of IL-6 and TNF-α.

### Safranal Inhibits MAPK and NF-κB Pathways in RAW264.7 Cells

**Figure 4:**
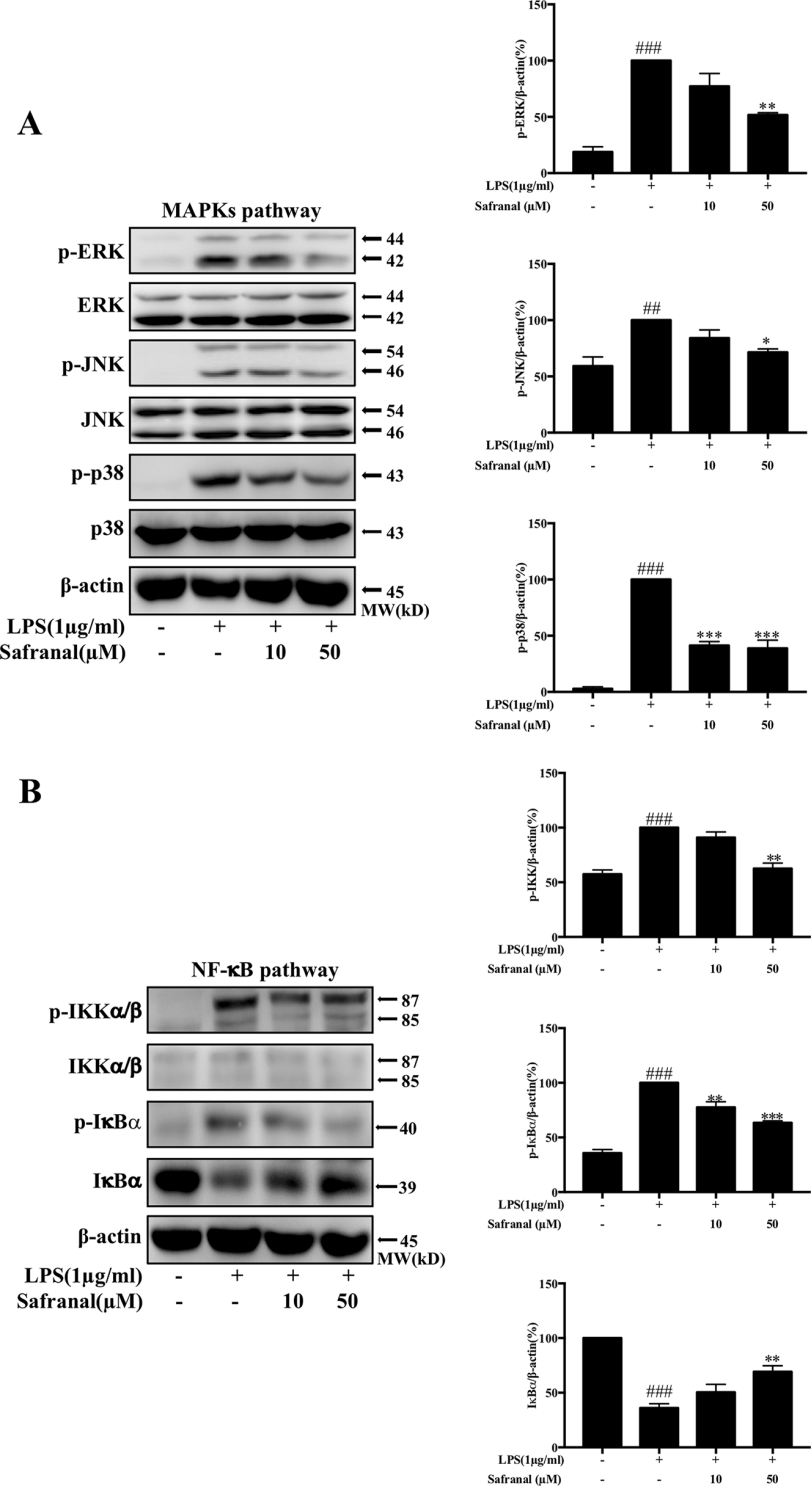
Safranal inhibits the NF-κB and MAPK pathways in lipopolysaccharide (LPS)-stimulated RAW264.7 cells. Cells were pretreated with Safranal for 1 h prior to LPS treatment. The levels of phosphorylated IKK, IκBα, ERK, JNK, and p38 were measured after treatment with 1 μg/ml LPS for 15 min. Signaling proteins in the **(A)** NF-κB pathway and **(B)** MAPK pathway were analyzed using Western blot and normalized to β-actin. The data shown are representative of three independent experiments and indicate the means ± SEM. ^###^*p* < 0.001 compared to non-treated group, *p < 0.05 compared to LPS, **p < 0.01 compared to LPS, ***p < 0.001 compared to LPS.

The activation of nuclear factor kappa-light-chain-enhancer of activated B cells (NF-κB) and mitogen-activated protein kinase (MAPK) pathways lead to the production of IL-6 and TNF-α. Therefore, we expected that Safranal would inhibit cytokine production via the inhibition of these signaling pathways. Western blot analysis results showed that Safranal inhibited the phosphorylation of MAPK pathway proteins extracellular signal-regulated kinase (ERK), c-Jun N-terminal kinase (JNK), and p38 (**Figure 4A**). Safranal also inhibited NF-κB pathway proteins IKKα/β and IκBα and the degradation of IκBα (**Figure 4B**).

### Safranal Inhibits Nuclear Translocation of NF-κB and AP-1 in RAW264.7 Cells

**Figure 5:**
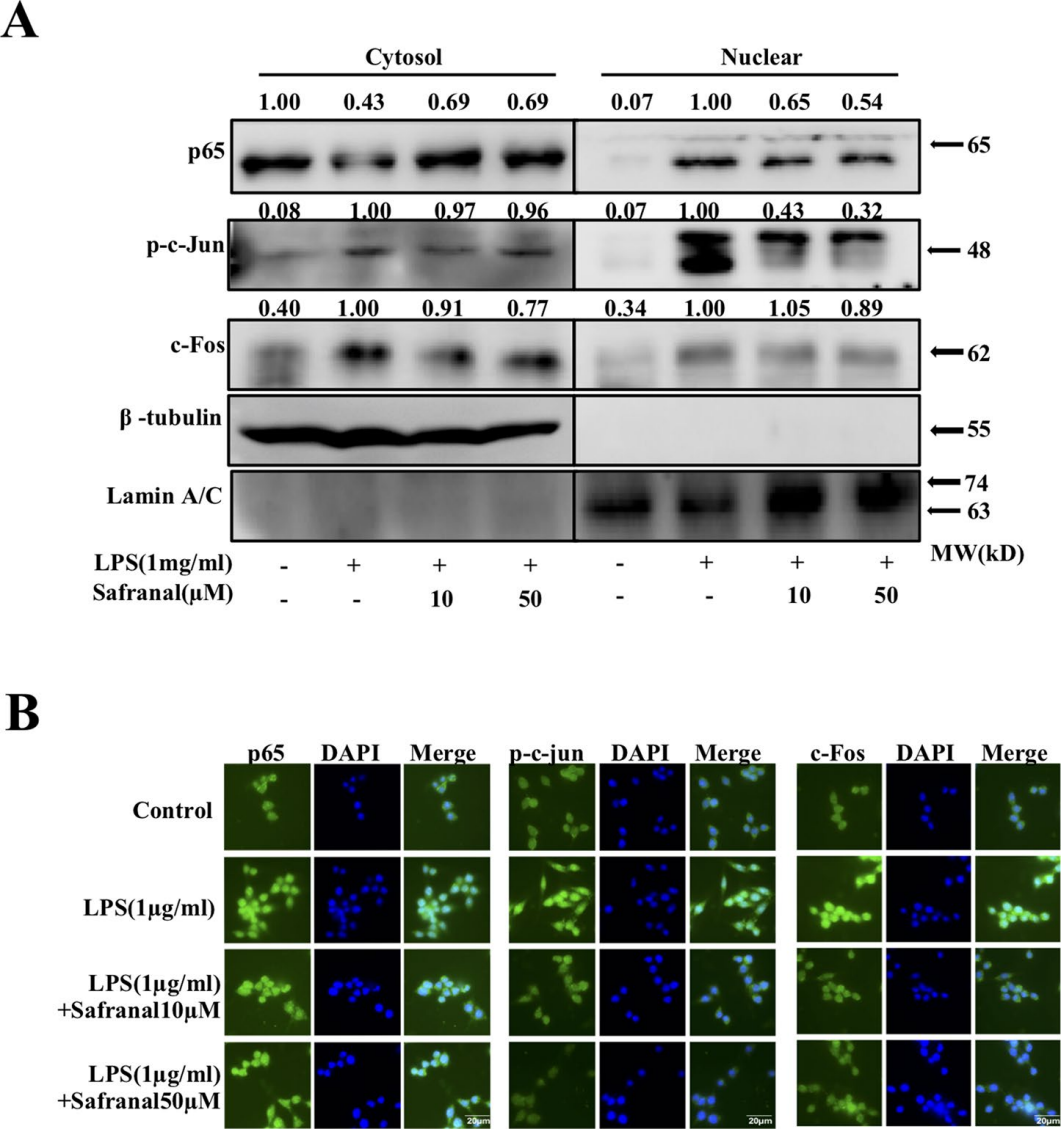
Safranal inhibits the nuclear translocation of NF-κB and AP-1 in lipopolysaccharide (LPS)-stimulated RAW264.7 cells. **(A)** Cells were collected after incubation with Safranal for 1 h followed by stimulation with 1 μg/ml of LPS for 30 min. Proteins in cytoplasmic or nuclear extracts from RAW264.7 cells were determined using Western blotting. RAW264.7 cells were seeded on coverslips overnight and incubated with Safranal prior to stimulation for 30 min. **(B)** Nuclear translocation of p65, p-c-Jun, and c-Fos were determined using immunofluorescence. The data shown are representative of three independent experiments. The scale labels shown are 20 µm.

Activated MAPK and NF-κB pathways lead to the nuclear translocation of NF-κB p65 and AP-1 and gene transcription, which leads to the production of cytokines. We further investigated whether Safranal inhibited the nuclear translocation of NF-κB p65 and AP-1 proteins. Stimulated RAW264.7 were collected, and nuclear and cytoplasmic fractions were analyzed using Western blot analyses. The results showed that Safranal inhibited the nuclear translocation of p65, p-c-Jun, and c-Fos proteins (**Figure 5A**). These results were confirmed using immunofluorescence (**Figure 5B**).

### Safranal Alleviated DSS-Induced Colitis Mice

**Figure 6:**
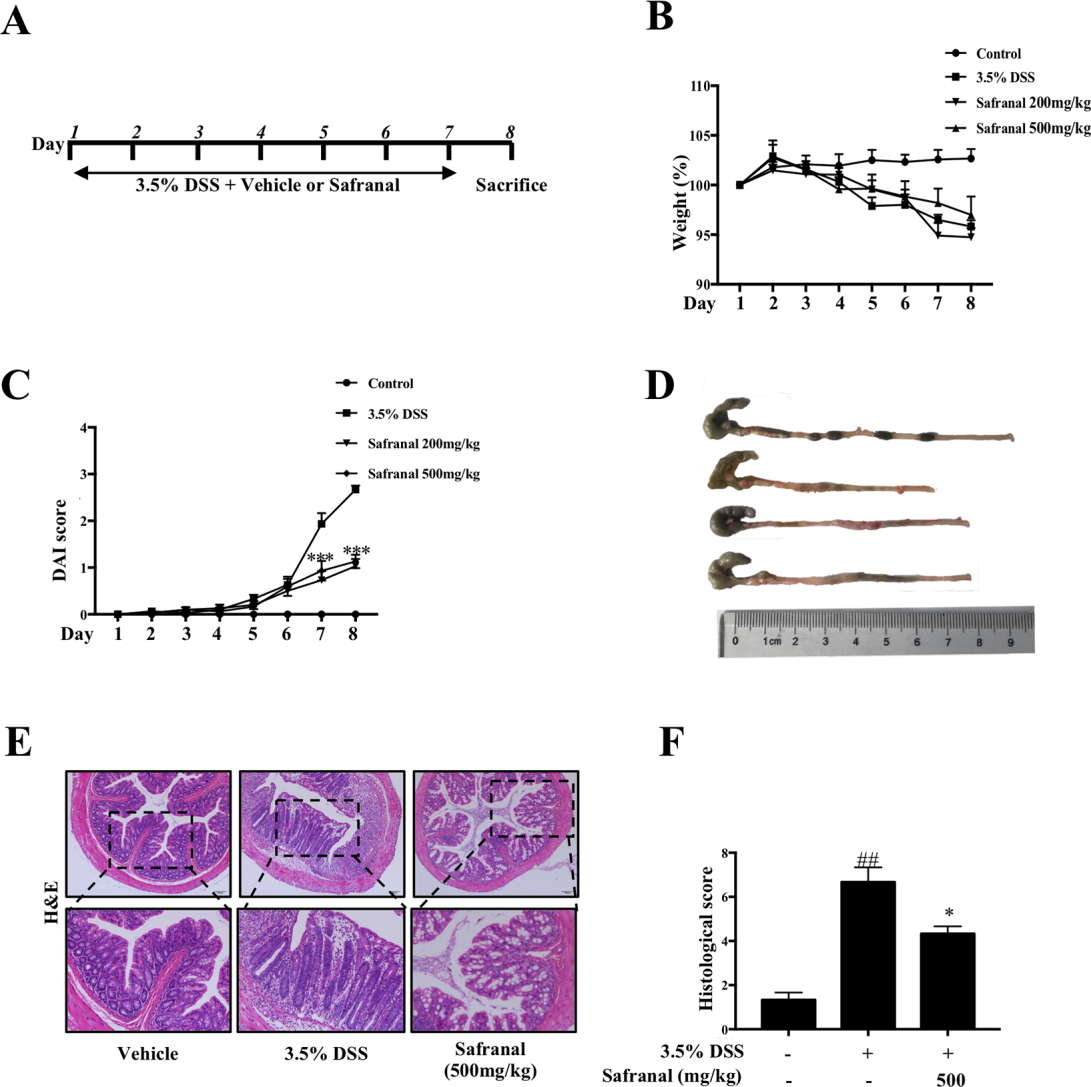
Safranal alleviates dextran sulfate sodium (DSS)-induced colitis mice. Mice were given DSS for 7 days with or without Safranal. Control group mice were given only vehicle **(A)**. Weight and DAI scores were recorded daily **(B**, **C)**. At the end of experiment, the colons were measured **(D)** and fixed in 4% paraformaldehyde for H&E staining **(E)**. The histological score was calculated **(F)**. All data shown are the means ± SEM of *n* = 10. ^##^*p* < 0.01 compared to non-treated group, *p < 0.05 compared to DSS-treated group, ***p < 0.001 compared to DSS-treated group. The scale labels shown are 100 µm.

UC is an inflammatory bowel disease in which common symptoms include weight loss, diarrhea, and loose and bloody stool. DAI scores, which are the sum of the percentage of weight loss, stool consistency, and rectal bleeding scores, were used to evaluate the clinical characteristics of DSS-induced colitis in mice. Mice were treated with 3.5% DSS with or without Safranal, and DAI scores were recorded daily (**Figures 6A**). Mice were sacrificed after 7 days, and colonic tissues, MLNs, and spleens were collected for further analyses. The percentage of weight loss and the colon length in the Safranal-treated groups were not significantly improved compared to the 3.5% DSS group (**Figures 6B, D**). However, we observed a slight restoration of colon length and percentage of weight loss, which suggest improvements in the Safranal-treated groups. We anticipated that increasing the length of Safranal administration may improve the outcomes. An increase in DAI score was observed on day 6 in all DSS-treated groups, indicating that this model was a competent model for colitis. On days 7 and 8, the DAI score of the Safranal-treated group was significantly lower than the 3.5% DSS group, suggesting that Safranal alleviated the clinical symptoms (**Figure 6C**).

UC is characterized pathologically by epithelial and crypt damage (Azad et al., 2019), resulting in impairment of intestinal barrier integrity. DSS stimulates UC as it damages crypts and goblet cells in colon tissues. H&E staining was used to examine the effect of Safranal on inflamed colon tissues. The results showed that Safranal decreased the severity of inflammation, depth of inflammatory involvement, and crypt damage (**Figures 6E, F**).

**Figure 7:**
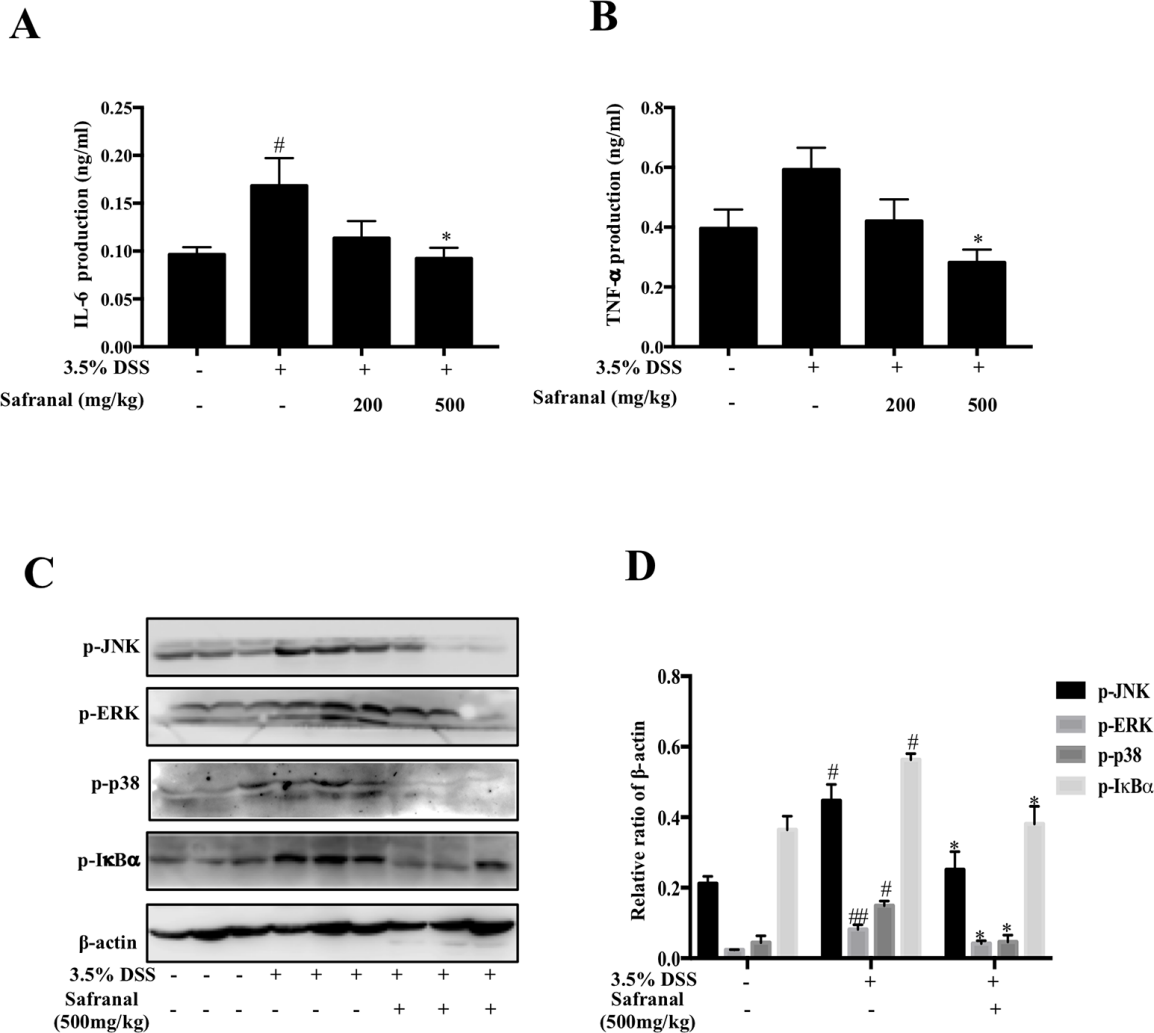
Safranal reduced cytokine production *via* suppression of MAPK and NF-κB proteins in colonic tissues from colitis mice. Colons were homogenized for measurement of IL-6 and TNF-α levels **(A**, **B)**. The phosphorylation of JNK, ERK, p38, and IκBα was analyzed using Western blotting **(C)** and the density of proteins was calculated and normalized to β-actin. All data are the means ± SEM, *n* = 10. ^#^p < 0.05 compared to- non-treated group ^##^p < 0.01 compared to non-treated group, *p < 0.05 compared to DSS-treated group. The scale labels shown are 100 µm.

Damaged epithelial cells interact with macrophages, which are the first responders to encounter gut microbes. Macrophages secrete pro-inflammatory mediators, such as IL-6 and TNF-α, which delay healing of colon epithelial cells (Khare et al., 2019). Safranal suppressed the production of IL-6 and TNF-α in colon tissue (**Figures 7A, B**). MAPK and NF-κB pathway proteins were analyzed, and Safranal inhibited ERK, JNK, p38, and IκBα phosphorylation (**Figure 7C, D**).

### Safranal Reduces the Accumulation of Macrophages in Colonic Tissue, MLNs, and Spleen

**Figure 8:**
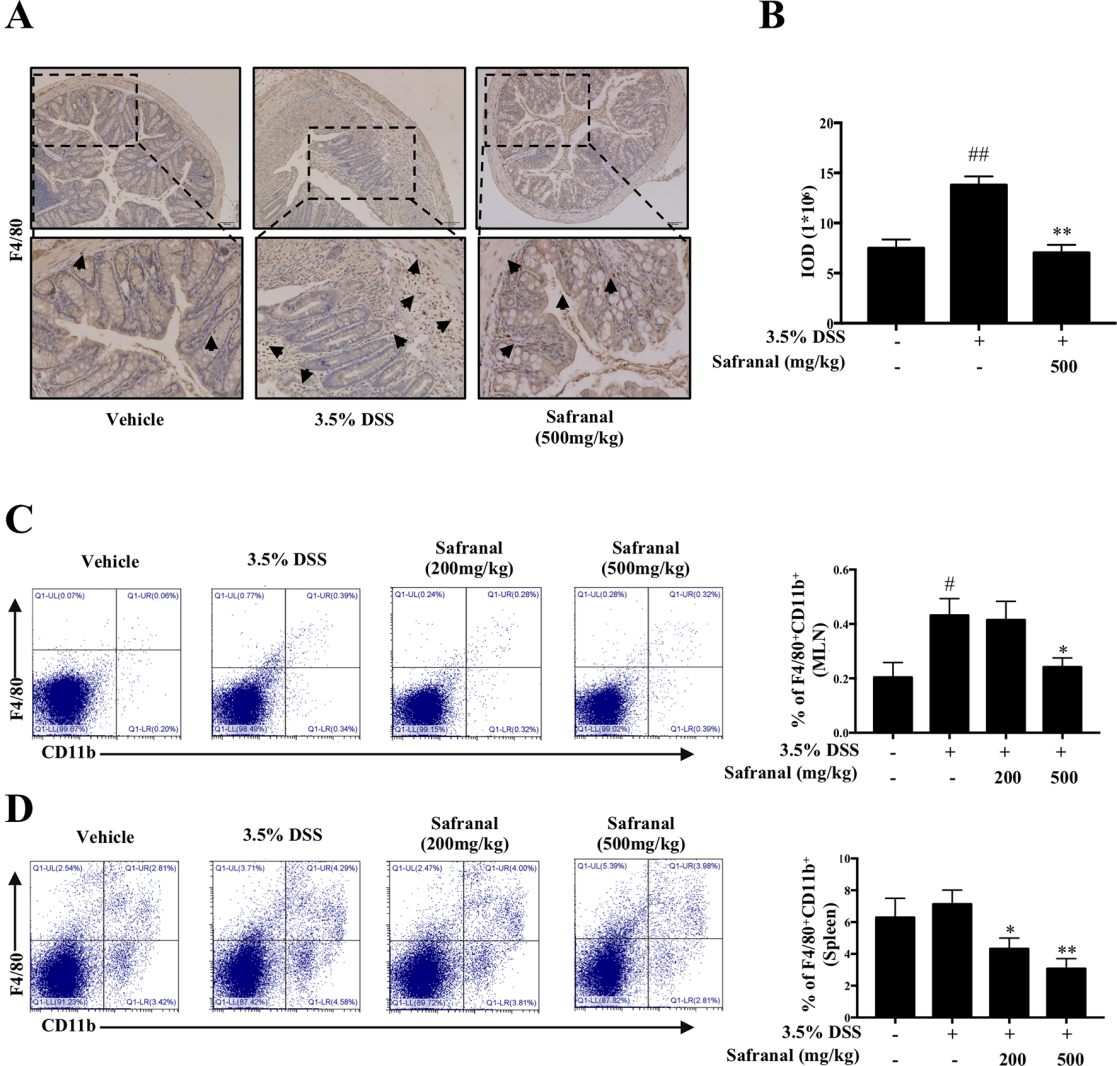
Safranal reduces macrophage infiltration in dextran sulfate sodium (DSS)-induced colitis mice. IHC of F4/80 was measured as an indication of macrophage infiltration in colon **(A)**. The value of integrated OD (IOD) were measured **(B)**. Mesenteric lymph nodes **(C)** and the spleen **(D)** were homogenized and visualized with antibodies following flow cytometry analyses. The data are presented as the means ± SEM of *n* = 10. ^#^*p* < 0.05 compared to non-treated group. ^##^*p* < 0.01 compared to non-treated group, *p < 0.05 compared to DSS-treated group, **p < 0.01 compared to DSS-treated group.

Macrophages play a major role in colonic inflammation. Therefore, we investigated the effect of Safranal on macrophage infiltration in colon tissues, MLNs, and spleen from colitis mice. IHC results showed a significant decrease in the infiltration of F4/80 macrophages in the Safranal-treated group compared to the 3.5% DSS group (**Figures 8A, B**). MLNs and spleen were homogenized, and monocytes were stained with F4/80 and CD11b antibodies to investigate the number of macrophages present in the tissues. The percentages of macrophages in the MLNs and spleens from DSS-induced colitis mice were detected by flow cytometry. The results showed that the amount of macrophages accumulated in MLNs and spleens were significantly decreased in Safranal-treated groups (**Figures 8C, D**).

## Discussion

Chinese herbal medicines have been used for the treatment of many diseases throughout history and play great roles in Chinese culture and health. Previous studies showed that compounds derived from Chinese herbs have beneficial effects on several diseases, such as thrombosis (Li et al., 2019a) and chronic heart failure (Han et al., 2018); thus, the discovery of new compounds derived from these herbs is crucial. As mentioned, first-line drugs produced effective results in the treatment of UC. However, some patients do not respond to these therapies, and other patients experience severe side effects. To overcome this problem, we found that many Chinese herbs exhibited therapeutic effects on colitis. Saffron is a commonly used herb in traditional Chinese medicine, and it is widely used in China as an anticoagulant and antidepressant agent. Clinical evidence shows the potential of saffron in the treatment of diseases. For example, saffron supplementation reduced the frequency of the clinical symptoms of asthma patients (Zilaee et al., 2019). Another study showed that saffron effectively alleviated mild and moderate depression compared to placebo (Toth et al., 2019). Saffron decreased cardiovascular risk factors, such as blood pressure, body weight, and waist circumference (Pourmasoumi et al., 2019). Although clinical trials seem to show significant results in many diseases, the active compounds and the mechanisms thereof require further investigation. Some research on compounds derived from saffron was performed *in vitro* and *in vivo*. Active compounds include Crocin, Crocetin, and Picrococin, which exhibit anti-inflammatory (Li et al., 2018), anticancer (Zhou et al., 2019), and antioxidant (Suh et al., 2019) effects. Therefore, we hypothesized that Safranal would affect colitis. The present study showed that Safranal alleviated DSS-induced colitis.

Macrophages play critical roles in many diseases, such as colitis (Wang et al., 2019) and airway inflammation (Kim et al., 2012). Macrophages are phagocytic immune cells that are substantial mediators in the innate immune system, and their functions include engulfing pathogens in their surroundings without being activated. However, some pathogens, such as LPS from Gram-negative bacteria, are recognized by Toll-like receptor 4 (TLR4) and initiate intracellular signaling, such as the two classical inflammatory-related pathways, MAPK and NF-ĸB (Hu et al., 2018). Phosphorylation of ERK, JNK, and p38 MAPKs activates activator protein 1 (AP-1), p-c-Jun, and c-Fos to translocate into the nucleus, which leads to gene expression (Ariyoshi et al., 2017). Activation of TLR4 leads to phosphorylation of the IKK complex in the NF-ĸB pathway, which leads to the phosphorylation and degradation of IκBα and the nuclear translocation of NF-ĸB p65. This dimer binds to DNA to facilitate gene transcription (Yuan et al., 2019). Transcription of AP-1 and NF-ĸB p65 leads to the production of pro-inflammatory mediators, such as IL-6 and TNF-α. Our data showed that Safranal decreased the production and mRNA levels of IL-6 and TNF-α in RAW264.7 cells. Western blot analysis data suggested that Safranal significantly suppressed the phosphorylation and expression of MAPK and NF-ĸB signaling pathway proteins. Safranal also inhibited the nuclear translocation of AP-1 and NF-ĸB p65. These results were confirmed in Western blot analyses of nucleic and cytoplasmic extracts and immunofluorescence. iNOS and COX-2 are two enzymes that participate in the production of NO and other inflammatory mediators. Safranal significantly inhibited iNOS, COX-2, and NO levels in RAW264.7 cells and primary BMDMs.

Macrophages produce pro-inflammatory mediators to promote vascular permeability and the recruitment of inflammatory cells, which enhances inflammatory responses. However, excess production of these cytokines leads to fever and septic shock. Some studies suggested that chronic inflammation enhanced the invasiveness of malignant cells (Binder et al., 2004). Therefore, inflammatory responses should not be left uncontrolled in many circumstances. Our data suggested that Safranal exhibited promising anti-inflammatory activity in macrophages, and we suggest the use of Safranal in other macrophage-mediated inflammatory diseases. Further studies on macrophage-related diseases, such as rheumatic arthritis, septic shock, asthma, and chronic inflammatory-related tumors, should be performed.

Macrophages are known to be responsible for the production of pro-inflammatory cytokines, and evidence showed that the reduction of pro-inflammatory macrophages in UC patients’ colon tissue significantly contributes to the therapeutic effect in UC patients (Zeissig et al., 2019). MLNs and spleen macrophages also play important roles in inflammatory diseases. MLNs are located on the wall of the large intestine and contain many types of monocytes. During colonic inflammation, the infiltration of macrophages increases in MLNs (Grunau et al., 2017) to assist the inflammatory response. Spleen macrophages participate in the elimination of pathogens during inflammation (Borges da Silva et al., 2015), and the number of macrophages infiltrating the spleen is also increased during inflammation. The present study focused on the effects of Safranal on macrophages, which are critical in the treatment of UC. Increasing numbers of macrophages infiltrate colon tissues in the DSS-induced colitis model (Song et al., 2019), which exhibits similar characteristics to human UC (Chassaing et al., 2014; Martin et al., 2016). Therefore, this colitis model was used in the present study. F4/80 and CD11b are two receptors on the macrophage cell membrane (Ahrens et al., 2008). We used antibodies targeting these proteins as indicators of macrophage presence in MLNs and the spleen. Flow cytometry analyses showed a decrease in the number of macrophages in Safranal-treated mice. IHC illustrated that Safranal significantly decreased the amount of F4/80^+^ macrophage infiltration in the colon.

The pathology of the UC colon includes damage to crypts, loss of goblet cells, depletion of the epithelial barrier, and leukocyte infiltration in the mucosa (Nunes et al., 2018). Clinical characteristics of UC include weight loss, abdominal pain, watery stools, and rectal bleeding. To duplicate the clinical and pathological characteristics of UC, a DSS-induced colitis model was used. The DAI score is the sum of the percentage of weight loss, stool consistency, and rectal bleeding scores, and it was used to evaluate the clinical severity of the animals. Histological evaluations suggested that Safranal reduced the severity of inflammation, crypt damage, and depth of inflammation.

On the molecular level, crypt damage in intestinal epithelium leads to interactions between intestinal Gram-negative microbes and immune cells, such as macrophages, which subsequently activate inflammatory-related receptors, such as TLR4 (Scarpa et al., 2011), and induce MAPK and NF-ĸB cell signaling cascades (Lee et al., 2011), which results in the production of pro-inflammatory cytokines, such as IL-6 and TNF-α(Pandurangan et al., 2016). These pro-inflammatory cytokines aggravate the inflammatory responses that affect epithelial healing process (Khare et al., 2019). Therefore, first-line drugs, such as TNF-α inhibitors, focus on reducing the production of these cytokines. Our results showed that colitis mice had lower levels of IL-6 and TNF-α after Safranal treatment than 3.5% DSS-treated mice, suggesting that Safranal alleviated the severity of colitis by decreasing IL-6 and TNF-α in colon tissue. These results indicate that Safranal alleviated colitis and the inhibition of macrophage-mediated inflammation.

## Conclusion

**Figure 9:**
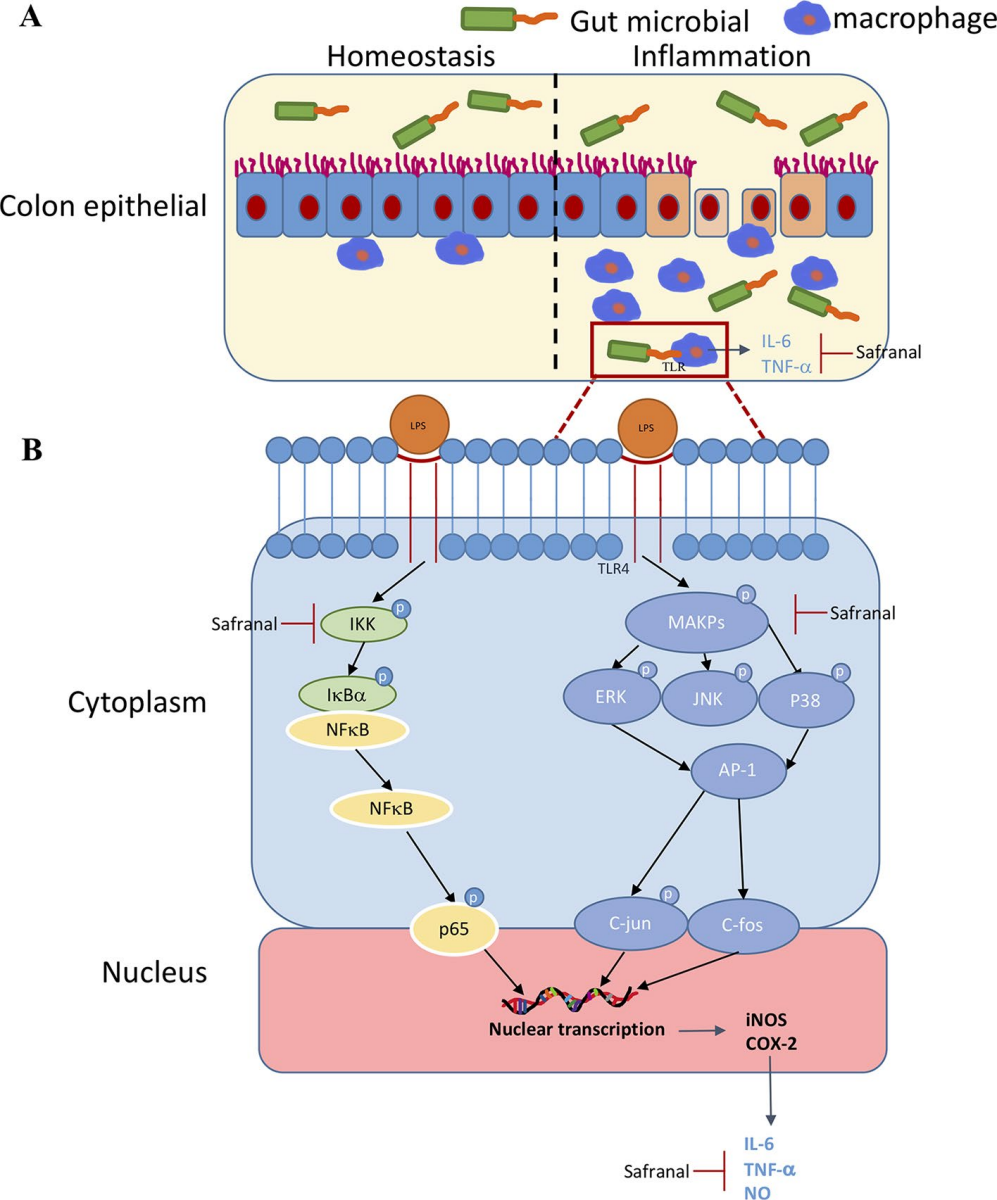
**(A)** Safranal alleviates dextran sulfate sodium-induced colitis. **(B)** Possible inhibitory mechanism of Safranal in lipopolysaccharide-stimulated macrophages.

In conclusion, our results showed the effect of Safranal in inhibiting macrophage-mediated inflammatory responses and alleviating DSS-induced colitis *via* inhibition of the MAPK and NF-ĸB signaling pathways (**Figures 9A, B**). These results provide a new therapeutic compound for the inhibition of macrophage-mediated inflammatory diseases, such as rheumatic arthritis, UC, cardiovascular disease, and septic complications.

## Data Availability Statement

All datasets generated for this study are included in the article/supplementary material.

## Ethics Statement

The animal ethics committee of Shanghai University of Traditional Chinese Medicine approved the animal experimental procedures and welfare (PZSHUTCM19011117).

## Author Contributions

HX, HZ, XZ, WP and YL supervised the project and acquired funding. PL collected and analyzed data and investigated the results. PL, YJ and NK collected specimens. HT, WF, and CZ reviewed the data and drafts of the manuscript.

## Funding

This work was financially sponsored by grants from the Shanghai Sailing Program (17YF1419500); the National Natural Science Foundation of China (NSFC) Grants 81803545 and 81602990; Professor of Special Appointment (Eastern Scholar) at Shanghai Institutions of Higher Learning; and the Three-Year Development Plan Project for Traditional Chinese Medicine (ZY(2018-2020)-CCCX-2001-02).

## Conflict of Interest

Authors XZ and WP were employed by company Shanghai Traditional Chinese Medicine Co., Ltd.

The remaining authors declare that the research was conducted in the absence of any commercial or financial relationships that could be construed as a potential conflict of interest.
